# Molecular characterization and serodiagnostic potential of *Echinococcus granulosus* hexokinase

**DOI:** 10.1186/s13071-021-04606-8

**Published:** 2021-02-08

**Authors:** Qi Xin, Miaomiao Yuan, Wei Lv, Huanping Li, Xiaoxia Song, Jun Lu, Tao Jing

**Affiliations:** 1 Institute of Pathogenic Biology, School of Basic Medical Sciences, Lanzhou University, Lanzhou, 730000 Gansu People’s Republic of China; 2 The Eighth Affiliated Hospital, Sun Yat-sen University, Shenzhen, 518000 Guangdong People’s Republic of China

**Keywords:** *Echinococcus granulosus*, Hexokinase, Immunolocalization, Immunogenicity, Indirect ELISA, Diagnosis

## Abstract

**Background:**

Cystic echinococcosis (CE), caused by the larval stage of *Echinococcus granulosus* (*sensu stricto*), is a life-threatening but neglected zoonosis. Glycolytic enzymes are crucial molecules for the survival and development of *E. granulosus*. The aim of this study was to investigate the molecular characterization, immunogenicity, tissue distribution and serodiagnostic potential of *E. granulosus* hexokinase (*Eg*HK), the first key enzyme in the glycolytic pathway.

**Methods:**

*Eg*HK was cloned and expressed in *Escherichia coli*. Specific serum antibodies were evaluated in mice immunized with recombinant *Eg*HK (r*Eg*HK). The location of *Eg*HK in the larval stage of *E. granulosus* was determined using fluorescence immunohistochemistry, and the potential of r*Eg*HK as a diagnostic antigen was investigated in patients with CE using indirect enzyme-linked immunosorbent assay (ELISA).

**Results:**

Recombinant *Eg*HK could be identified in the sera of patients with CE and in mouse anti-r*Eg*HK sera. High titers of specific immunoglobulin G were induced in mice after immunization with r*Eg*HK. *Eg*HK was mainly located in the tegument, suckers and hooklets of protoscoleces and in the germinal layer and laminated layer of the cyst wall. The sensitivity and specificity of the r*Eg*HK-ELISA reached 91.3% (42/46) and 87.8% (43/49), respectively.

**Conclusions:**

We have characterized the sequence, structure and location of *Eg*HK and investigated the immunoreactivity, immunogenicity and serodiagnostic potential of r*Eg*HK. Our results suggest that* Eg*HK may be a promising candidate for the development of vaccines against *E. granulosus* and an effective antigen for the diagnosis of human CE.
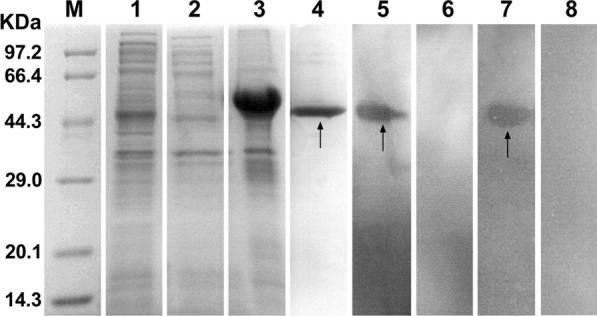

## Background

Cystic echinococcosis (CE) is caused by the larval stage of *Echinococcus granulosus* (*sensu stricto*) and occurs globally in livestock husbandry areas of South America, North Africa, Australia, western, central and eastern Europe and central Asia, particularly in western China [1]. CE is a life-threatening but neglected zoonosis, especially in developing countries, that causes severe disorders of serious public health and economic concern [2]. A global estimate suggests that at least 50 million humans are infected with *E. granulosus*, with approximately more than 170,000 new cases every year [3, 4], resulting in an estimated 285,000 (95% confidence interval: 218,515–366,133) disability-adjusted life years lost per annum and an annual economic loss of approximately US$3 billion [3].

Similar to other parasites, the larval stage of *E. granulosus* obtains glucose from their hosts as their energy source. Analysis of *E. granulosus* genome data demonstrated that *E. granulosus* have complete pathways for both glycolysis and the tricarboxylic acid cycle during infection [5], while glycolysis is the main pathway for *E. granulosus* to generate energy and the vital intermediate products for physiological metabolism [6, 7]. Clearly, glycolytic enzymes play a crucial role in *E. granulosus* survival. To date, various glycolytic enzymes of *E. granulosus*, such as fructose-bisphosphate aldolase, enolase [8] and triosephosphate isomerase [9], have been identified in the tegument and parenchyma tissue of the parasite; these show antigenic properties and potential multifuncionality in *E. granulosus*. Thus, glycolytic enzymes represent promising targets for the development of both immune and drug intervention measures against echinococcosis.

Hexokinase (HK) (ATP: D-hexose-6-phosphotransferase, EC 2.7.1.1.) is an enzyme that facilitates the first step in glycolysis and catalyzes the phosphorylation of glucose to produce glucose 6-phosphate. HK is an important enzyme of glycolysis [10], and HK isozymes are widely distributed in a wide variety of species, ranging from plants, microbes, parasites to mammals including humans. The characteristics and functions of HK have been well examined in many parasites to date, including *Plasmodium falciparum* [11–13], *Leishmania mexicana* [14], *Trypanosoma brucei* [15, 16], *Trypanosoma cruzi* [17–19], *Haemonchus contortus* [20], *Brugia malayi* [21], *Schistosoma mansoni* [22–24] and *Clonorchis sinensis* [25, 26], but available information on HK from *E. granulosus* (*Eg*HK) is still limited.

In this study, the sequence and structure of *Eg*HK were analyzed. A recombinant *Eg*HK (r*Eg*HK) was expressed and the location of *Eg*HK in the larval stage of *E. granulosus* was determined using fluorescence immunohistochemistry. Additionally, the serodiagnostic potential of the r*Eg*HK was investigated in patients with CE. Our results represent the first experimental data of HK in *E. granulosus* and provide the foundation for further studies on *Eg*HK in the framework of the diagnosis and prevention of human CE.

## Methods

### Ethics statement

Animal care and management procedures were conducted in compliance with the guidelines of the Institutional Animal Caring and Using Committee of Lanzhou University. Animals had free access to water and commercial mouse chow throughout the study. The experiment involving human participants was approved by the Human Research Ethics Committee of Lanzhou University. Each participant was provided with an explanation of the nature of the research and the study protocol, following which they all signed the informed consents.

### Bioinformatic analysis

The complementary DNA (cDNA) sequence encoding *Eg*HK was obtained from the cDNA library of adult *E. granulosus* constructed by our laboratory. The DNA sequence translations and the predictions of protein molecular mass, isoelectric point (pI), conserved domains and protein properties of *Eg*HK were performed with ExPASy (http://au.expasy.org/tool/pi-tool.html). The TMHMM Server v.2.0 (http://www.cbs.dtu.dk/services/TMHMM/) was used to predict the transmembrane domain and active center. SignalP 5.0 (http://www.cbs.dtu.dk/services/SignalP/) and SecretomeP 2.0a (http://www.cbs.dtu.dk/services/SecretomeP/) were used to predict the signal peptide. The B-cell linear epitopes were analyzed using BepiPred software (version 1.0). The amino acid sequences of HK from different species were obtained from the GenBank and GeneDB databases. Multiple sequence alignment was performed using Clustal X software (version 2.0). The phylogenetic tree was constructed by the neighbor-joining method using MEGA software (version 7).

### Construction, expression and purification of r*Eg*HK

The forward and backward primers were designed according to the open reading frame of the full-length nucleotide sequence of *Eg*HK. The coding sequence of *Eg*HK was PCR-amplified using the forward primers GAGGATCCATGGGGGTGCAATTC and backward primers CAGTCGACCTAGCCCGCGAAAAC with the BamHI and SalI restriction enzyme sites (underlined), respectively. The generated fragments were directionally inserted into the corresponding multiple cloning sites of pET30a(+) to construct plasmids encoding *Eg*HK. After verification of the DNA inserts by sequencing, the recombinant plasmids were transformed into *Escherichia coli* strain BL21 (DE3) for the expression of *Eg*HK. After induction with 0.2 mM isopropyl β-d-thiogalactopyranoside for 7 h at 20 °C, the cells were harvested and resuspended in a buffer containing 50 mM NaH_2_PO_4_ and 300 mM NaCL and lysed by sonication. The samples were then sedimented by centrifugation at 12,000 *g* for 10 min at 4 °C to collect the inclusion bodies which were subsequently dissolved in solubilization buffer (8 M urea and 50 mM Tris, pH 8.0) at a ratio of 1:10 (w/v) for 30 min at 4 °C and then refolded by gradient dialysis for 60 h at 4 °C. The purification of the his-tagged r*Eg*HK was performed using a Ni^2+^ affinity chromatography column (Qiagen, Hilden, Germany), and the molecular mass and purity of the purified proteins were assessed by 12% (v/v) sodium dodecyl sulfate-polyacrylamide gel electrophoresis (SDS-PAGE). The protein concentration was determined with a BCA Protein Assay Kit (Beyotime, Shanghai, China).

### Immunization of mice with r*Eg*HK

Eight-week-old specific pathogen-free female BALB/c mice (18–20 g) were obtained from the Laboratory Animal Center of Lanzhou University. The mice were immunized subcutaneously with 50 μg purified r*Eg*HK emulsified in an equal volume of complete Freund’s adjuvant (Sigma-Aldrich, St. Louis, MO, USA), followed up by two boosts with 50 μg protein in an equal volume of incomplete Freund’s adjuvant (Sigma-Aldrich) at 2-week intervals. The mice in the control group received the same inoculation as the r*Eg*HK-immunized group with only difference in the process being the replacement of r*Eg*HK with phosphate buffered saline (PBS). The immune sera were then collected and identified by enzyme-linked immunosorbent assay (ELISA) for antibody titer, using horseradish peroxidase-conjugated goat anti-mouse immunoglobulin G (IgG).

### Western blotting

A 10-μg aliquot of purified r*Eg*HK were transferred from the 12% SDS-PAGE gel to a PVDF membrane. The membrane was blocked with 5% skim milk in Tris-buffer saline-Tween 20 (TBST) overnight and then incubated with mouse anti-r*Eg*HK sera, sera collected from patients with CE, sera collected from healthy subjects or pre-immune mouse sera, at a dilution of 1:200 at 4 °C overnight. After two washes with TBS, the membrane was incubated with horseradish peroxidase-conjugated goat anti-mouse IgG or goat anti-human IgG (1:2000 dilution) (Sigma-Aldrich) at 37 °C for 1 h. The immunoblots were detected using an Enhanced Chemiluminescence Substrate Kit (Thermo Fisher Scientific, Waltham, MA, USA).

### Indirect immunofluorescence assays

*Echinococcus granulosus* protoscoleces and cyst walls were isolated aseptically from hydatid cysts removed from the liver of infected sheep slaughtered in an abattoir (Xining, Qinghai, China) and fixed in 4% paraformaldehyde for 20 h. Cyst walls were embedded in paraffin, sliced into 3-μm-thick sections, deparaffinized in xylene, dehydrated in ethanol and then incubated with 0.01 M citrate buffer at 95 °C for 30 min for thermal remediation. After three washes with PBS, the tissues were blocked with 5% bovine serum albumin (BSA) in PBS and treated with mouse anti-r*Eg*HK sera or pre-immune mouse sera at a dilution of 1:200, at 37 °C for 1 h, respectively, following which fluorescein isothiocyanate-conjugated goat anti-mouse IgG (1:200 dilution) (Sigma-Aldrich) was added and incubated at 37 °C for a further 1 h. Fluorescence was detected and images were acquired on an immunofluorescence microscope (model IX71; Olympus Corp., Tokyo, Japan ).

### r*Eg*HK ELISA

Sera from patients with CE (46 samples) and patients with *Taenia solium* cysticercosis (26 samples) were obtained from the Institute of Pathogenic Biology, Lanzhou University, China. Sera from healthy students (23 samples), collected at Lanzhou University, were used as a control group. Microplates were coated with 100 μL/well of r*Eg*HK at 5 μg/ml in coating buffer overnight at 4 °C. After three washes with PBST, the microplates were incubated with 1% BSA at 37 °C for 1 h for blocking. The serum samples were then added at 1:200 dilution in 1% BSA in PBS–Tween (PBST) and incubated at 37 °C for 1 h. The second antibody (horseradish peroxidase-conjugated goat anti-human IgG; Sigma-Aldrich) was diluted 1:5000 in 1% BSA in PBST and incubated at 37 °C for 1 h. Subsequently, TMB substrate buffer was added (100 μL/well) and incubated at 37 °C for 15 min. Finally, the reaction was terminated with 2 M H_2_SO_4_ (50 μL/well) and the absorbance was read at 450 nm. All sera were tested in duplicate. The cut-off value was determined by the mean OD_450_ value of the 23 healthy sera sample plus two standard deviations. The sensitivity (%) of the method was determined by the percentage value of ELISA positive samples and true positive cases, while the specificity (%) was determined by that of ELISA negative samples and true negative cases. Sera from patients with cysticercosis (26 samples) were used to determine the cross-reactivity of r*Eg*HK.

### Statistics analysis

SPSS 19.0 software (IBM Corp., Armonk, NY, USA) was used for statistics analysis. The ELISA serological result was analysed using the nonparametric Kruskal-Wallis H-test. The results were considered statistically significant at *P* < 0.05.

## Results

### Sequencing and bioinformatics analysis of *Eg*HK

**Fig. 1.**
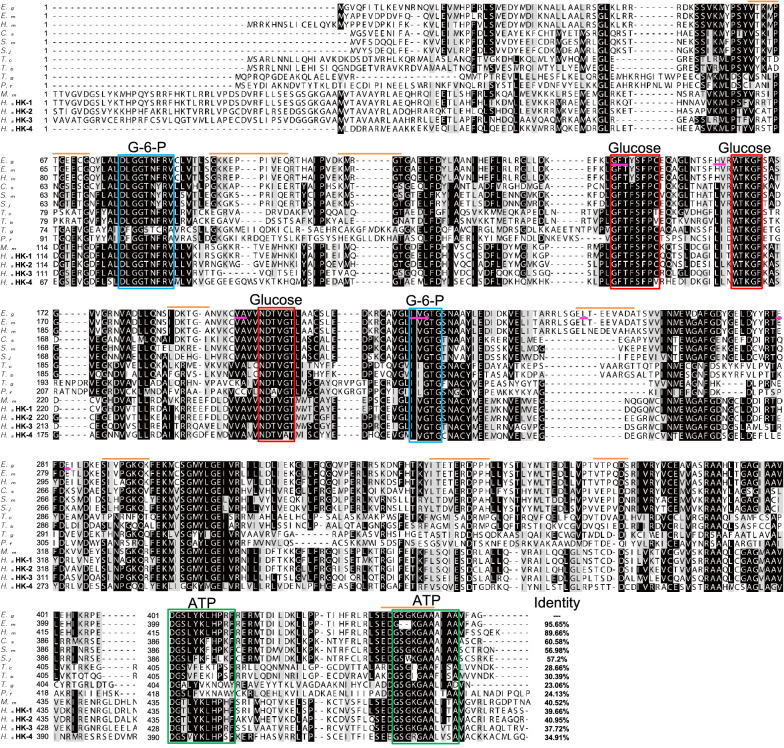
The amino acid sequence alignment analysis of *Echinococcus granulosus* hexokinase (*Eg*HK) with the homologous hexokinase (HK) from other parasite species: *Echinococcus granulosus* (*E. g*, MW292450), *Echinococcus multiloculari* (*E. m*, CDS38325.1), *Hymenolepis microstomia* (*H. m*, CDS34381.1 ), *Clonorchis sinensis* (*C. s*, GAA52956.1), *Schistosoma mansoni* (*S. m*, AAA29894.2), *Schistosoma japonicum* (*S. j*, CAX74187.1), *Trypanosoma cruzi* (*T. c*, AAL93565.1), *Trypanosoma brucei* (*T. b*, CAC69958.1), *Toxoplasma gondii* (*T. g*, BAB55664.1), *Plasmodium falciparum* (*P. f*, ETW53097.1), *Mus musculus* (*M. m,* AAB57759.1), *Homo sapiens* HK-1 (*H. s* HK-1, NP000179.2), *Homo sapiens* HK-2 (*H. s* HK-2, NP000180.2), *Homo sapiens* HK-3 (*H. s* HK-3, NP002106.2), *Homo sapiens* HK-4 (*H. s* HK-4, AAH01890.1). In the amino acid sequence, the conserved domains for glucose, glucose 6-phosphate (*G-6-P*) and adenosine triphosphate (*ATP*) are illustrated in red, blue and green rectangles, respectively. The letters marked with pink underlines are putative glucose binding sites (144aa, 145aa, 146aa, 161aa, 162aa, 196aa, 197aa, 217aa, 221aa, 222aa, 223aa, 249aa, 280aa, 283aa). Nine potential B-cell epitopes (ep1:62–72 aa; ep2:93–102 aa; ep3:111–115 aa; ep4:186–191 aa; ep5:248–256 aa; ep6:289–296 aa; ep7:343–352 aa; ep8:370–375 aa; ep9:448–456 aa) are marked with orange overlines

The full-length cDNA sequence of *Eg*HK comprised 1395 nucleotides encoding 464 amino acids (aa). The predicted molecular weight and pI was 51.7 kDa and 6.12, respectively. The instability index was 38.72, lower than the threshold value (40), indicating that *Eg*HK was a stable protein. There was no predicted transmembrane region in the deduced amino acid sequence. Analysis by SignalP showed that the deduced protein had no signal peptide. *Eg*HK had a highly conserved active polypeptide chain (LGFTYSFPCEQAGLNTSFHVRWTKGF, 143–168 aa), which is the signature acid sequence of HK [27] (Fig. 1). There were 17 putative B-cell linear epitopes in the amino acid sequence of *Eg*HK, nine of which (ep1:62–72 aa; ep2:93–102 aa; ep3:111–115 aa; ep4:186–191 aa; ep5:248–256 aa; ep6:289–296 aa; ep7:343–352 aa; ep8:370–375 aa; ep9:448–456 aa), according to the comparison with the homologous human B-cell epitopes, suggested promising diagnostic and vaccine potentials. The putative glucose binding sites involve the following amino acids: Ser144, Phe145, Pro146, Thr161, Lys162, Asn196, Asp197, Leu217, Gly221, Thr222, Asn223, Glu249, Gln280 and Glu283 [27] (Fig. 1), all of which are conserved in *Eg*HK.

### Homologous sequence alignment

**Fig. 2.**
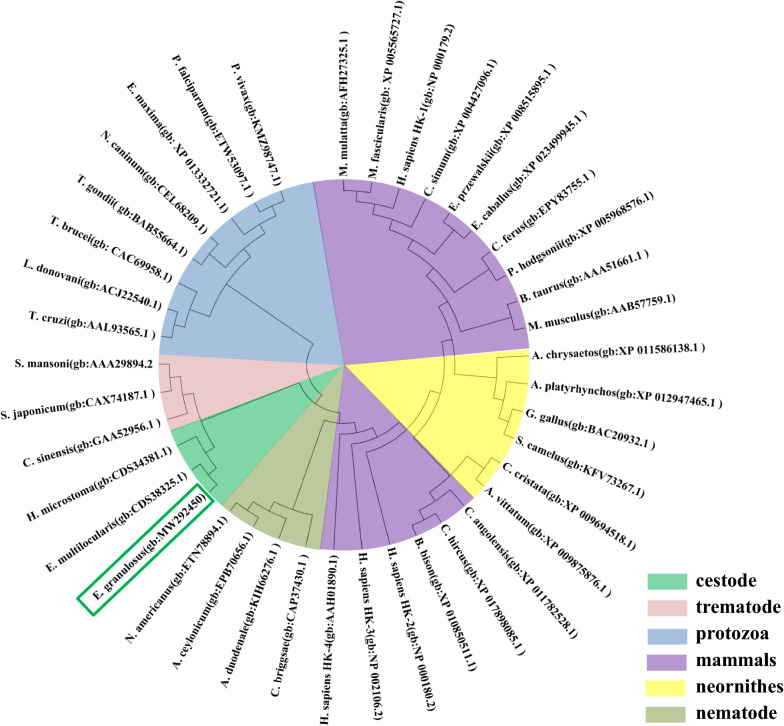
The phylogenetic analysis of *Eg*HK with homologous HK. The phylogenetic tree was constructed using the neighbor-joining method. *E. granulosus*, *Echinococcus granulosus*; *E. multilocularis*, *Echinococcus multilocularis*; *H. microstoma*, *Hymenolepis microstomia*; *C. sinensis*, *Clonorchis sinensis*; *S. japonicum*, *Schistosoma japonicum*; *S. mansoni*, *Schistosoma mansoni*; *T. cruzi*, *Trypanosoma cruzi*; *L. donovani*, *Leishmania donovani*; *T. brucei*, *Trypanosoma brucei*; *T. gondii*, *Toxoplasma gondii*; *N. caninum*, *Neospora caninum*; *E. maxima*, *Eimeria maxima*; *P. falciparum*, *Plasmodium falciparum*; *P. vivax*, *plasmodium vivax*; *M. mulatta*, *Macaca mulatta*; *M. fascicularis*, *Macaca fascicularis*; *C. simum*, *Ceratotherium simum*; *E. przewalskii*, *Equus przewalskii*; *E. caballus*, *Equus caballus*; *C. ferus*, *Camelus ferus*; *P. hodgsonii*, *Pantholops hodgsonii*; *B. taurus*, *Bos Taurus*; *M. musculus*, *Mus musculus*; *A. chrysaetos*, *Aquila chrysaetos*; *A. platyrhynchos*, *Anas platyrhynchos*; *G. gallus*, *Gallus gallus*; *S. camelus*, *Struthio camelus*; *C. cristata*, *Cariama cristata*; *A. vittatum*, *Apaloderma vittatum*; *C. angolensis*, *Colobus angolensis*; *C. hircus*, *Capra hircus*; *B. bison*, *Bison bison*; *H. sapiens*, *Homo sapiens*; *C. briggsae*, *Caenorhabditis briggsae*; *A. duodenale*, *Ancylostoma duodenale*; *A. ceylanicum*, *Ancylostoma ceylonicum*; *N. americanus*, *Necator americanus. gb* GenBank ID

Homologous sequence alignment revealed that *Eg*HK shared 95.65% identity with HK from *Echinococcus multilocularis* (*Em*HK), 89.66% identity with *Hymenolepis microstomia* (*Hm*HK), 60.58% identity with *C. sinensis* (*Cs*HK) and 56.98% identity with *S. mansoni* (*Sm*HK). Moreover, *Eg*HK shared 39.68, 40.95, 37.72 and 34.91% identity with *Homo sapiens* HK-1, HK-2, HK-3 and HK-4, respectively. The phylogenetic tree revealed that *Eg*HK was clustered into a branch closely related to HK from other cestode parasites, such as *E. multilocularis* and *H. microstomia*, and had the closest genetic relationship with *E. multilocularis* (Fig. 2).

### Expression, purification and identification of r*Eg*HK

**Fig. 3.**
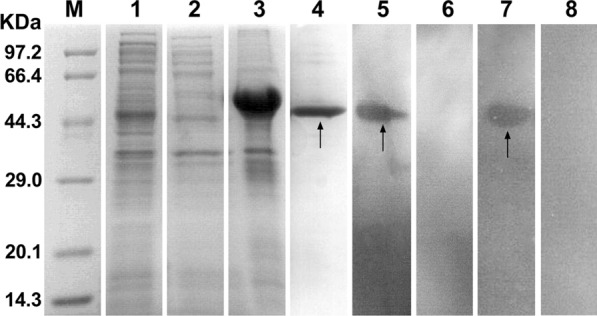
Expression, purification and western blot analysis of r*Eg*HK. Lanes:* M* Molecular marker (in KDa),* 1* induced *E. coli* BL21 (DE3) transformed with pET30a,* 2* soluble supernatant fraction of induced *E. coli* BL21 (DE3) transformed with pET30a-*Eg*HK,* 3* pellet fraction of induced *E. coli* BL21 (DE3) transformed with pET30a-*Eg*HK,* 4* purified r*Eg*HK,* 5* purified r*Eg*HK probed with anti-r*Eg*HK sera,* 6* purified r*Eg*HK probed with pre-immunized sera,* 7* purified r*Eg*HK probed with sera from patient with CE,* 8* purified r*Eg*HK probed with sera from healthy subjects

*Eg*HK was expressed as an insoluble protein and existed in an inclusion body in *E. coli* BL21 (DE3) as a His-tag protein (Fig. 3). Purified r*Eg*HK was detected by SDS-PAGE and approximately presented the expected molecular weight of 51.7 kDa. The anti-sera against r*Eg*HK exhibited a high titer (1:51,200). Recombinant *Eg*HK was recognized by mouse anti-sera against r*Eg*HK and the sera from patients with CE (Fig. 3), while it was not recognized by pre-immune mouse sera or sera from healthy subjects.

### Immunolocalization of *Eg*HK in larval stage of *E. granulosus*

**Fig. 4.**
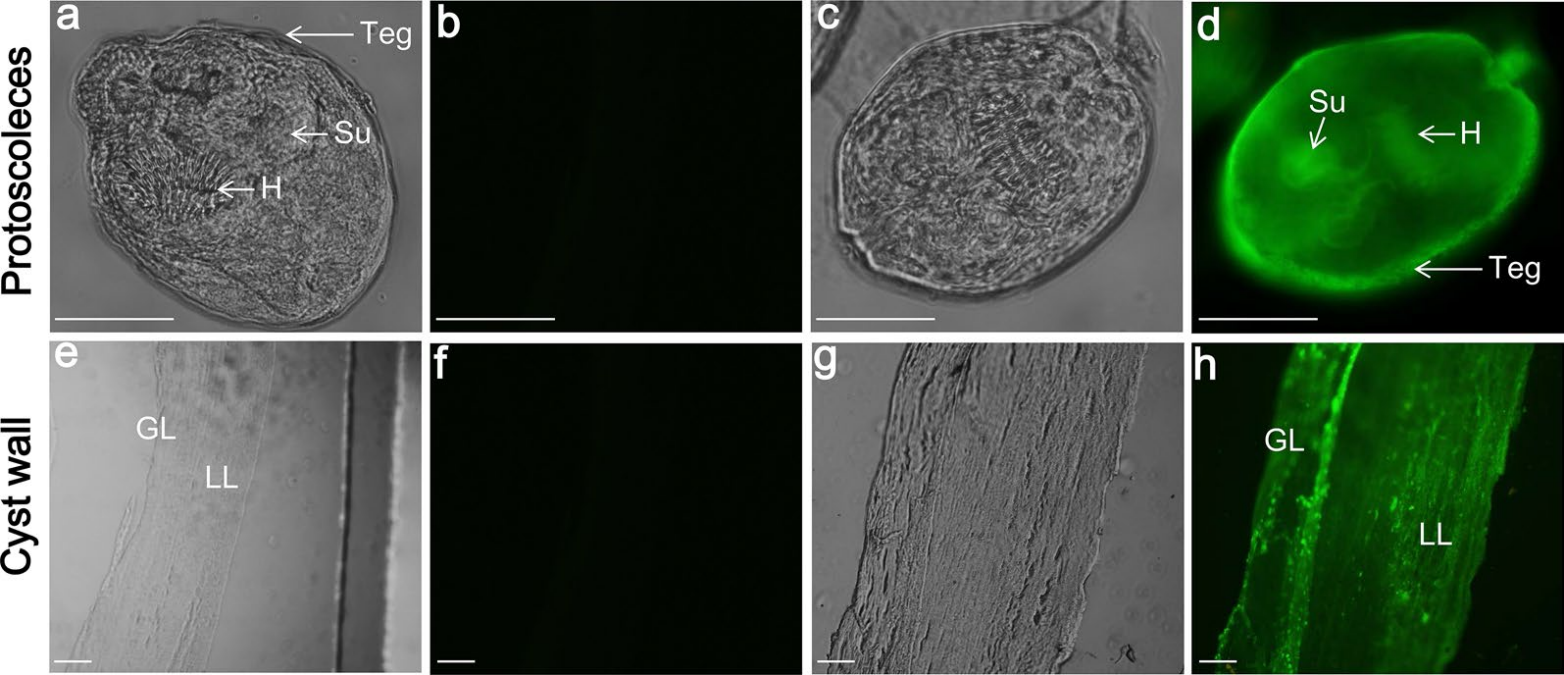
Immunolocalization of *Eg*HK in larval stage of *E. granulosus*. *Eg*HK in the protoscoleces and the cyst walls was immunofluorescently labeled using mouse anti-r*Eg*HK sera as the primary antibody, followed by fluorescein isothiocyanate-conjugated anti-mouse immunoglobulin G as secondary antibody. Sera from pre-immune mice were applied as primary antibody for a negative control.** b**,** d**,** f**,** h** are fluorescence microscopy images;** a**,** c**,** e**,** g** are the respective images of the same samples observed under white light.* Teg* Tegument,* Su* sucker,* H* hooklet,* LL* laminated layer,* GL* germinal layer. Scale bars: 50 μm

To detect the localization of *Eg*HK in the larval stage of *E. granulosus*, immunofluorescence assay was performed on *E. granulosus* protoscoleces and cyst walls using mouse anti-r*Eg*HK sera. The results showed strong fluorescence intensity on the tegument surface, suckers and hooklets of protoscoleces (Fig. 4). Also, the entire cyst wall, including the germinal layer (GL) and laminated layer (LL), exhibited green fluorescence. No specific fluorescence was detected in the negative controls.

### Serology assay by r*Eg*HK

**Fig. 5.**
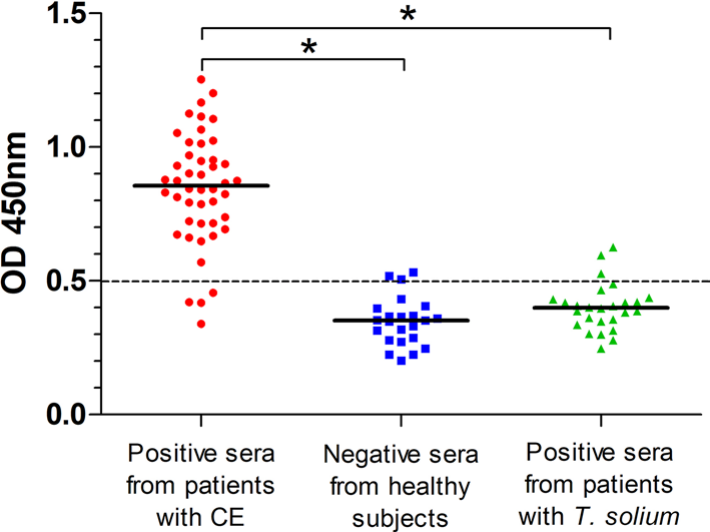
Serological results from the enzyme-linked immunosorbent assay for sera from patients with cystic echinococcosis (*CE*) or *Taenia solium* cysticercosis and from healthy, negative subjects for *Eg*HK. Horizontal black lines represent the median values. Dashed horizontal line indicates the cut-off value (0.5). Asterisk indicates a signficant difference at *P* < 0.0001.

To evaluate the serodiagnostic potential of r*Eg*HK, the sera of patients with CE or *Taenia solium* cysticercosis and of healthy subjects were analyzed by indirect ELISA. The cut-off value calculated from the 23 samples collected from healthy subjects was 0.5 (Fig. 5). Forty-two serum samples from patients with CE were detected as positive for r*Eg*HK, indicating a sensitivity of 91.3% (42/46). The OD_450_ values of serum samples from patients with CE were significantly higher than those of patients with cysticercosis or healthy subjects (Kruskal-Wallis H-test:* χ*^2^ = 63.571,* df* = 2, *P* < 0.0001). The OD_450_ values of 20 serum samples from the healthy subjects and 23 samples from patients with cysticercosis were lower than the cut-off value, indicating a specificity of 87.8% (43/49) in the assay. Moreover, three sera out of 26 samples from cysticercosis patients displayed cross-reactivity with r*Eg*HK.

## Discussion

In the study reported here, we analyzed the sequence, structure, immunoreactivity, immunogenicity and tissue distribution of *Eg*HK in the larval stage of *E. granulosus*. We also evaluated the serodiagnostic potential of *Eg*HK in CE patients.

In mammals, the HK family consists of three 100-kDa isozymes (HK-1, HK-2, and HK-3) and one 50-kDa isozyme (HK-4), also referred to as glucokinase. However, non-mamalian organisms generally have a 50-kDa HK. It has been postulated that the 100-kDa hexokinases evolved through the duplication and tandem ligation of a glucokinase-like gene that encodes an ancestral 50-kDa HK [28]. The analysis of the amino acid squence showed that there was a single form of HK in *E. granulosus* and that *Eg*HK possesses the typical characteristics of a non-mammalian HK (Fig.1). When subjected to SDS-PAGE, the molecular mass of r*Eg*HK was approximately 51.7 kDa (Fig. 3), which is the same as that described for HK from other parasites, such as *P. falciparum*, *S. mansoni*, and *C. sinensis* [11, 22, 25].

As the first key enzyme in the glycolytic pathway, HK begins glycolysis and catalyzes the phosphorylation of glucose to produce glucose 6-phosphate. Previous gene expression profile has shown that HK is consistantly expressed in the oncospheres and in the larval and adult stages of *E. granulosus*. Furthermore, HK is upregulated in protoscoleces, the hydatid cyst and especially in adult worms compared with oncospheres, suggesting that HK plays important roles in controlling and maintaining stage-specific features of the parasite during its life-cycle [5]. Thus, the crucial importance of glycolysis to *E. granulosus* and the lower identity (34.91%) of *Eg*HK with human glucokinase (HK-4) suggest that this molecule could be a promising target for both chemotherapy and vaccine development.

The immunofluorescence study demonstrated that *Eg*HK is extensively distributed in protoscoleces and the cyst wall, where protoscoleces are formed, suggesting that as a key glycolytic enzyme, *Eg*HK plays a vital role in the formation of the GL and the growth of protoscoleces. This conclusion is also supported by previous observations of high levels of *Eg*HK mRNA and protein in the larval stage of *E. granulosus* [5, 29]. In protoscoleces, in particular, *Eg*HK was observed to be abundantly expressed in the tegument. It is well known that the tegument of cestode is closely associated with the absorption of nutrients. Thus, the distribution of *Eg*HK in the tegument indicates its pivotal role in the absorption and digestion of glucose from the host for energy supply. Furthermore, the localization of *Eg*HK in the suckers and hooklets of protoscoleces might be associated with the energy requirement for the process of attaching to the host intestine. The cyst wall, which is composed of the GL and LL, is the parasite–host interface and is permeable; as such, it is believed to be involved in the parasite–host interaction, enabling the diffusion of macromolecules of at least 150 kDa [30]. The outer, acellular LL, in particular, is widely regarded as being a crucial element of parasite–host interplay [31]. Given that *Eg*HK was detected in not only the GL, but also in the acellular LL, the localization of *Eg*HK in the metacestode suggests that *Eg*HK might be a component of excretory/secretory (ES) products and mediate direct interaction with host cells.

In our study, the high titer of anti-r*Eg*HK antibody IgG demonstrates the strong immunogenicity of r*Eg*HK. In addition, r*Eg*HK was recognized by anti-r*Eg*HK mouse sera and the sera from patients with CE, demonstrating its good immunoreactivity. In particular, r*Eg*HK recognition by the sera from patients with CE suggests that *Eg*HK might be a component of ES products of the *E. granulosus* metacestode, which is consistent with previous conjectures. However, the predicted amino acid sequence of *Eg*HK contains no signal peptide or transmembrane domain. Thus, we speculate that *Eg*HK may rely on the release of exosomes, a special protein secretion pathway that has been reported in *E. granulosus* [32], for extracellular secretion.

At present, the commercially available serological kits mostly use hydatid cyst fluid (HCF) as diagnostic antigen, collected from infected animals. However, the complex and heterogeneous composition of HCF negatively impacts on the sensitivity and specificity of the tests [33, 34]. Furthermore, there is a growing evidence indicating that recombinant proteins and high-purity synthetic antigen preparations are more reliable for serodiagnostic application than native antigens. Thus, research on recombinant proteins and synthetic peptides is an important strategy to develop more sensitive and specific tests [35]. As the first enzyme in glycolysis, *Eg*HK, which is crucial to *E. granulosus*, has been identified as a potential antigenic protein [29]. Therefore, in this study, we evaluated the diagnostic value of r*Eg*HK in patients with CE using an indirect ELISA. The sensitivity and specificity of the r*Eg*HK assay achieved 91.3% and 87.8%, respectively, in the pilot serological assay, reflecting its potential applications. Nevertheless, r*Eg*HK showed cross-reactions with three serum samples out of 26 samples of sera from patients with *T. solium* cysticercosis, indicating that *Eg*HK protein shares a few of the same or similar epitopes with *T. solium* proteins. This is inevitable considering their close genetic relationship. Analysis of the cross-reaction of r*Eg*HK with *E. multilocularis* was not performed due to the lack of serum samples from alveolar echinococcosis patients. Therefore, further studies are needed to validate the clinical applicability of r*Eg*HK in the future.

### Conclusions

We report here the bioinformatic characterization of *Eg*HK. Immunofluorescence assay verified that *Eg*HK is mainly expressed on the tegument, suckers and hooklets of protoscoleces and on the cyst wall. The recombinant *Eg*HK showed relatively good immunogenicity and immunoreactivity. The indirect r*Eg*HK-ELISA indicated a good sensitivity (91.3%) and specificity (87.8%) for the detection of antibodies in sera from patients with CE. Our results suggest that *Eg*HK may be a promising candidate for development of vaccines against *E. granulosus* and an effective antigen for the diagnosis of human CE*.*

## Data Availability

The full-length DNA sequence of *Eg*HK has been deposited in GenBank database (https://www.ncbi.nlm.nih.gov/) under the accession number MW292450. Data supporting the conclusions of this article are included within the article. The data used and/or analyzed during the current study are available from the corresponding author upon reasonable request.

## Abbreviations

CE: cystic echinococcosis
HK: hexokinase
*Eg*HK: *E. granulosus* HK
r*Eg*HK: recombinant *Eg*HK
cDNA: complementary DNA
aa: amino acids
GL: germinal layer
LL: laminated layer
ELISA: enzyme linked immunosorbent assay
